# Population Pharmacokinetics of Piperacillin in Non-Critically Ill Patients with Bacteremia Caused by Enterobacteriaceae

**DOI:** 10.3390/antibiotics10040348

**Published:** 2021-03-25

**Authors:** Vicente Merino-Bohórquez, Fernando Docobo-Pérez, Adoración Valiente-Méndez, Mercedes Delgado-Valverde, Manuel Cameán, William W. Hope, Álvaro Pascual, Jesús Rodríguez-Baño

**Affiliations:** 1 Unidad de Gestión de Farmacia Hospitalaria, Hospital Universitario Virgen Macarena, 41009 Sevilla, Spain; vicente.merino.sspa@juntadeandalucia.es (V.M.-B.); manuel.camean.sspa@juntadeandalucia.es (M.C.); 2 Departamento de Farmacología, Universidad de Sevilla, 41009 Sevilla, Spain; 3 Departamento de Microbiología, Universidad de Sevilla, 41009 Sevilla, Spain; apascual@us.es; 4 Instituto de Biomedicina de Sevilla IBIS, Hospital Universitario Virgen Macarena/CSIC/Universidad de Sevilla, 41013 Sevilla, Spain; adoracion.valiente.sspa@juntadeandalucia.es (A.V.-M.); mercdss@gmail.com (M.D.-V.); jesusrb@us.es (J.R.-B.); 5 Red Española de Investigación en Patología Infecciosa (REIPI RD16/0016), Instituto de Salud Carlos III, 28029 Madrid, Spain; 6 Unidad Clínica de Enfermedades Infecciosas, Microbiología y Medicina Preventiva, Hospital Universitario Virgen Macarena, 41009 Sevilla, Spain; 7 Department of Molecular and Clinical Pharmacology, University of Liverpool, Liverpool L69 3GE, UK; william.hope@liverpool.ac.uk; 8 Royal Liverpool and Broadgreen University Hospital Trust, Liverpool L69 3GE, UK; 9 Departamento de Medicina, Universidad de Sevilla, 41009 Sevilla, Spain

**Keywords:** bloodstream infection, renal function, neurotoxicity, nephrotoxicity, pharmacokinetics, piperacillin–tazobactam, Enterobacteriaceae

## Abstract

This study analyzes the pharmacokinetic variability of piperacillin in non-critically ill patients with Enterobacteriaceae bloodstream infections (EBSI) and explores predicted clinical outcomes and piperacillin-related neurotoxicity under different renal conditions. Hospitalized, non-critically ill patients treated with piperacillin–tazobactam for EBSI were included. Four serum samples per patient were collected and analyzed. A population pharmacokinetic model was developed using the Pmetrics package for R. Monte Carlo simulations of various dosage regimens of 4 g piperacillin, administered q8 h or q12 h by short (0.5 h) or long (4 h) infusion, following the different glomerular filtration rate (GFR) categories used to classify chronic kidney disease (Kidney Disease: Improving Global Outcomes, KDIGO) to determine the probability of target attainment (PTA) using a free drug concentrations above the minimal inhibitory concentration (*f*T > MIC) of 50% for efficacy and targets for piperacillin-associated neurotoxicity. Twenty-seven patients (102 samples) were included. Extended piperacillin infusions reached a PTA > 90% (50%*f*T > MIC) within the susceptibility range, although a loading dose did not greatly improve the expected outcome. Long infusions reduced the expected toxicity in patients with severe renal impairment. The study supports the use of extended infusions of piperacillin in non-critically ill patients with EBSI. No benefits of a loading dose were expected in our population. Finally, extended infusions may reduce the risk of toxicity in patients with severe renal impairment.

## 1. Introduction

Enterobacteriaceae infections are associated with increased morbidity and mortality [1,2,3], which is mainly related to delay in providing active therapy, together with the lower efficacy of certain alternative drugs compared with first-line antibiotics. In this respect, appropriate initial antimicrobial therapy is of key importance for the resolution of serious infections. Several studies have demonstrated that inappropriate empiric therapy is independently associated with a worse outcome in patients with bacteremia caused by Enterobacteriaceae [1,4]. 

Piperacillin is a beta-lactam antibiotic, commonly combined with tazobactam, with a broad spectrum of bactericidal activity against Gram-negative and Gram-positive aerobes and anaerobes [5], and is frequently used for the empiric treatment of severe infections potentially caused by extended-spectrum beta-lactamase (ESBL)-producing Enterobacteriaceae and *Pseudomonas aeruginosa* [5]. Beta-lactam activity is related to the fraction of the dosing interval during which free drug concentrations remain above the minimal inhibitory concentration (*f*T > MIC) for the relevant pathogen [6]. In the case of piperacillin, an *f*T > MIC of at least 50% of the dosing interval is considered necessary for maximal activity [7]. Most studies included in systematic reviews and meta-analyses across a wide range of severely ill patients admitted to intensive care units (ICU) found significant benefits in terms of mortality and clinical cure associated with prolonged versus intermittent infusions of piperacillin–tazobactam (TZP) [8,9]. Despite these obvious benefits, a survey on antimicrobial dosing in the ICU found wide variability in reported practices for TZP dosing and monitoring [10]. While there is an extensive published literature on piperacillin pharmacokinetics (PK) concerning variability in plasma concentrations and strategies aimed at optimizing efficacy in critically ill patients [11,12,13], there is scant information relating to non-critically ill patients. Piperacillin has been found to be safe and well tolerated in many clinical trials [14]. Even so, recent studies have shown neurological deterioration related to high piperacillin serum concentrations in a range from 11.4% to 43.3% and nephrotoxicity in 8.5% of non-ICU and ICU patients [15,16]. Two different papers have suggested pharmacokinetic targets associated with piperacillin-related neurotoxicity. Quinton et al. found that a serum concentration threshold of 157.2 mg/L is predictive of the occurrence of piperacillin neurotoxicity, [15] while Imani et al. found that a piperacillin C_min_ > 361.4 mg/L or > 452.65 mg/L was associated with a 50% risk of developing a neurotoxicity or nephrotoxicity event, respectively [16]. 

The aim of this prospective study was to describe the population PK of piperacillin in a cohort of non-critically ill patients with bloodstream infection (BSI) due to Enterobacteriaceae and to perform Monte Carlo simulations to explore the safety and expected outcomes of different dosing regimens suitable for non-critically ill patients.

## 2. Results

### 2.1. Patients

A total of 27 non-critically ill patients treated with piperacillin–tazobactam for Enterobacteriaceae BSI were included. Demographic, epidemiological, clinical, and microbiological variables and the outcomes of episodes are shown in Table 1. Three patients with creatinine clearance (CrCl) < 20 mL/min/1.73 m^2^ received 4/0.5 g every 12 h. A total of 102 plasma samples were collected at steady state during a single dosing interval. None of the determinations were below the limit of quantification. The most common source of bacteremia was the urinary tract (66.7%). *Escherichia coli* and *Klebsiella pneumoniae* were the most frequently isolated microorganisms. The piperacillin–tazobactam MIC was ≤8 mg/L in 24 patients. One patient died (3.7%), and no adverse events related to piperacillin–tazobactam administration were noted.

### 2.2. Pharmacokinetic Model

The piperacillin concentration–time data were best described by the one-compartment linear model, and no significant reduction in the log-likelihood value was observed compared with the two- or three-compartment models. The inclusion of creatinine clearance (CrCl) as a covariate best described drug clearance (CL) and reduced the log-likelihood value by 152 points. No other covariates improved the final model. The ordinary differential Equation (1) for the model is the following:

(1) $$dC/dt=R\left(1\right)-(\left(Intercept+Slope\;\times ClCr)/V_{c}\right)\times X_{1}$$

**Figure 1:**
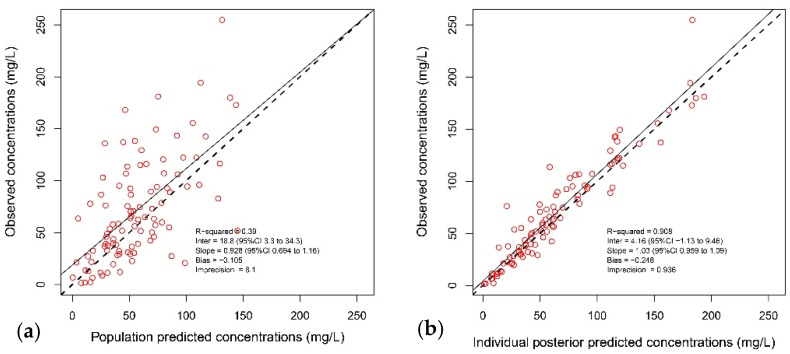
Diagnostic plots of the final population pharmacokinetic covariate model. (**a**) Observed piperacillin concentrations versus population predicted concentration (R^2^ = 0.39); (**b**) observed piperacillin concentrations versus individual predicted concentrations (R^2^ = 0.908). The continuous line represents the regression line, and the dashed line is the line of identity.

*X*_1_ is the amounts of piperacillin (in milligrams) in the central and peripheral compartment, respectively. Intercept and slope are the coefficients of a linear relationship of piperacillin clearance versus ClCr. The final parameter estimates from the population model are presented in Table 2, and the observed-versus-individual predicted piperacillin concentrations appear in Figure 1.

**Figure 2:**
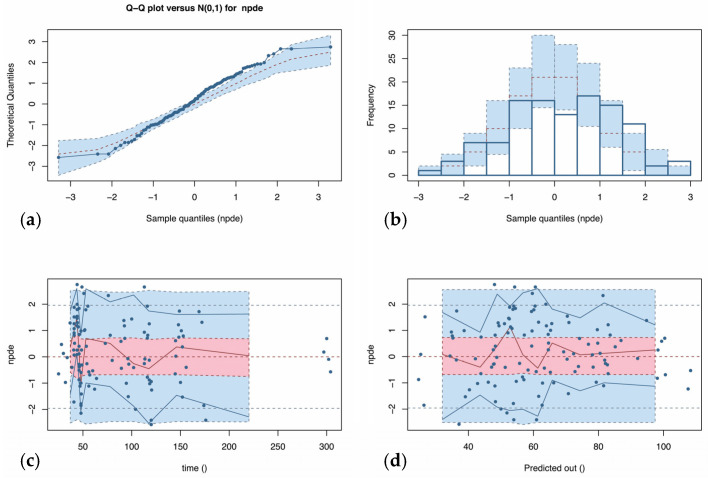
Normalized distribution predicted error (NPDE) versus predicted piperacillin concentration (**a**) and time (**b**). The NPDE should be a standard normal distribution with a mean of 0 and a standard deviation of 1, i.e., ~N (0,1). The approximately normal distribution of NPDE points, as indicated by the quantile–quantile (Q-Q) plot (**c**) and the NPDE histogram (**d**), centered at 0, indicates that the population model predictions are minimally systematically biased. The horizontal dashed lines in the top left and top right panels are at −2, 0 (the mean), and +2 standard deviations for the ideal normal distribution, and the surrounding error of 95% is shown with gray boxes. The solid horizontal lines are the actual distributions of the NPDE. The histogram columns in the bottom right panel compare the actual frequencies of NPDE with the ideal normal distribution (dashed line).

**Figure 3:**
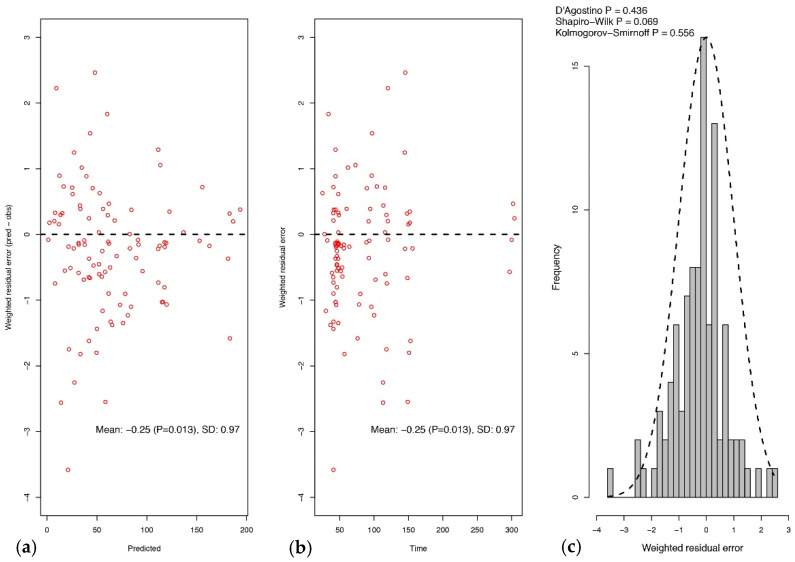
Weighted residual error plot (population predicted versus observed concentrations, mg/L) versus population predictions (**a**) and time of observation (**b**) and frequency distribution of weighted residual errors (**c**).

The results of the normalized distribution prediction error (NPDE) analysis (Q-Q plot and histogram) are summarized graphically in Figure 2. The weighted residual error distributions are shown in Figure 3. The NPDEs (*p* = 0.069 in the Shapiro–Wilk test of normality), weighted residual error distributions, and visual predictive check (VPC) plot (Figure 2) suggest that the fit of the model to the data was acceptable.

### 2.3. Probability of Target Attainment

The Monte Carlo simulations of varying piperacillin regimens and the probabilities of target attainment (PTAs) for > 50% *f*T > MIC are shown in Table 3. The results showed that the lower PTA (*f*T *>* MIC for 50% of the dosing interval) was associated with a higher ClCr. Piperacillin dosages of 4/0.5 g (4 h infusion) q8 h or first dose [4/0.5 g (0.5 h infusion) + 4 g (4 h infusion)] followed by 4/0.5 g (4 h infusion) q8 h reached a PTA of >90% within the current breakpoint (i.e., piperacillin MIC ≤ 8 mg/L). In this analysis, no significant benefit of a loading dose was observed. An extended infusion, alone or given with a loading dose, showed slightly a better PTA than the short piperacillin infusion.

Table 3 also shows the PTAs for >50% *f*T > MIC with different dosage regimen adjustments and levels of creatinine clearance. With a dosage adjustment of 4/0.5 g (0.5 h infusion) q12 h, patients with severely reduced renal function or kidney failure achieved the optimal targets of *f*T > MIC > 50% over the dosing interval within the susceptible range. A prolonged infusion administration (4 h) improved the PTA within the intermediate category compared with a short one, and similar results were found with the addition of a loading dose.

### 2.4. Piperacillin Neurotoxicity and Nephrotoxicity

At the simulated doses of 4/0.5 g (4 h infusion) q8 h or first dose [4/0.5 g (0.5 h infusion) + 4 g (4 h infusion)] followed by 4/0.5 g (4 h infusion) q8 h in patients with normal renal function, no neurotoxicity or nephrotoxicity events were expected. With respect to the case with dose adjustments of piperacillin due to renal disease, the simulated patients did not reach the nephrotoxicity breakpoint of C_min_ > 452.65 mg/L. Also, in these simulated group patients with severely decreased renal clearance or kidney failure, no neurotoxicity in those who received short-term intravenous infusions was predicted. However, long infusions or the addition of a loading dose slightly increased the risk of expected neurotoxicity to 0.2% and 0.3%, respectively (targeting at C_min_ > 157.2 mg/L).

## 3. Discussion

The present study describes the population pharmacokinetics of piperacillin targeted at non-critically ill patients. We focused on PTA (50% *f*T > MIC) based on some traditional animal and human studies, although more demanding targets are used for critical patients, as the patients in our study were not critically ill [17,18,19]. We observed that only creatinine clearance significantly affected the pharmacokinetics of piperacillin. We also observed that an extended infusion of piperacillin improved the PTA (50% *f*T > MIC). Our data show that the currently suggested dosing regimen (4/0.5 g infusion over 30 min q8 h) is less likely to attain the pharmacodynamic target in patients with normal or severe renal impairment. Other investigators have previously reported similar results in both critically and non-critically ill patients [20,21,22].

The administration of piperacillin by extended infusion, over half the dosing interval (i.e., infused over 4 h, administered q8 h), or after an initial loading dose helped overcome subtherapeutic piperacillin drug exposures in all the simulated scenarios. This has previously been shown in observational studies as well as randomized controlled trials (RCT) and also correlates with the higher rates of clinical cure in the RCTs [23,24,25]. In our model, we observed a slight benefit with the administration of a loading dose, especially against a piperacillin MIC of 32 mg/L (considered as resistant). We cannot rule out the possibility of an extended benefit in patients with augmented renal clearance (>130 mL/min/1.73 m^2^) or in patients with other baseline characteristics. These results differ somewhat with those of Rhodes et al. [26], who suggested that loading dosages may be necessary for their modeled dosing schedules of piperacillin–tazobactam by prolonged infusion (3.375 g infused over 4 h) and continuous infusion (10.125 g infused over 24 h) and for elevated MICs. However, in that study, the authors only analyzed the probability of and time to first instance of concentrations exceeding the breakpoint MIC for piperacillin–tazobactam (16 mg/L, Clinical and Laboratory Standard Institute [CLSI] breakpoints) over the first 120 min of therapy. It is also important to note that a different TZP dosage was used (3 g piperacillin plus 0.375 g tazobactam instead of 4 g piperacillin plus 0.5 g tazobactam).

Piperacillin-associated neurotoxicity in patients with dose adjustments due to impaired glomerular filtration rates was also analyzed. Piperacillin-associated neurotoxicity was not observed in our patients in this study, and simulations of extended infusions showed a probability of <1% (targeting C_min_ > 361 mg/L), which contrasts with the probability of 11.4% observed by Imani et al. [16]. However, in the latter study, piperacillin dosages of 8–16 g/24 h at 6–12 h intervals were included. No information regarding dosage adjustment in patients with renal impairment was given, and this could explain the higher frequency found if piperacillin concentrations were not correctly adjusted. On the other hand, Quinton et al. analyzed a retrospective cohort of ICU patients [15], which showed a piperacillin-associated neurotoxicity of 43.4%, using a piperacillin concentration equal to or higher than 157.2 mg/L as a target. Applying this breakpoint to our study, overall, we predict no neurotoxicity events in patients with normal renal function and a probability of 0.2–0.3% in those with adjusted doses (dosage 5, 4 g (4 h infusion) q12 h and dosage 6, loading dose of 4 g (0.5 h infusion) + 4 g (4 h infusion) followed by 4 g (4 h infusion) q12 h) due to severe renal impairment. Nevertheless, the study by Quinton et al. was not intended to estimate the proportion of piperacillin-associated neurotoxicity, and such a frequency would probably be overestimated.

With regard to the absence of piperacillin-related nephrotoxicity observed in our patients with normal renal function or impaired renal function with adjusted dose, our results fit with our predicted nephrotoxicity event based on the target of C_min_ > 452.65 mg/L [16]. However, Imani et al. showed nephrotoxicity events in 8.5% of the study population following exclusion criteria, which is higher than that observed in our patients. In this study, the use of a diuretic agent was associated with a significantly increased risk of piperacillin nephrotoxicity (OR 31.32, IC95% 3.33–294.70, *p* < 0.01). Apart from other baseline patient characteristics between both studies, the administration of this diuretic agent consumption was not taken into account in Imani’s study. Thus, we cannot compare this important factor associated with nephrotoxicity with our population.

Although our findings highlight the importance of optimal dosing and administration of piperacillin based on renal clearance in non-critically ill patients in order to overcome subtherapeutic concentrations, only 27 patients were included in the present study, which limits the generalizability of these results. Additionally, the model was largely constructed using patients with normal renal function and few with severe renal impairment, which could impair the extrapolation of results to patients with extreme renal impairment. The PK/PD target for predicting piperacillin efficacy also remains unclear. While the majority of centers currently performing β-lactam therapeutic drug monitoring (TDM) target values of 100% *f*T > MIC, others use 100% *f*T > 4 × MIC, 50% *f*T > 4 × MIC, or 70% *f*T > 4 × MIC for certain β-lactams. Whether serum concentrations of β-lactams always need to be above the MIC requires prospective clinical validation [27]. Finally, because of the major differences in the expected piperacillin-associated neurotoxicity and nephrotoxicity based on the choice of published pharmacokinetic targets, the results should be interpreted with caution until they have been prospectively validated.

## 4. Materials and Methods

### 4.1. Study Design and Patients

A prospective study was conducted at the Hospital Universitario Virgen Macarena (Seville, Spain) between October 2012 and February 2015, including adult patients (age > 17 years) with BSI caused by Enterobacteriaceae who received initial monotherapy with piperacillin–tazobactam. Eligible patients were first identified from daily reports of blood cultures from the microbiology laboratory. Patients were included only if: the first piperacillin–tazobactam dose was administered in the first 12 h after collection of blood cultures, the duration of the therapy was at least 48 h, and the patient was not admitted to the intensive care unit. Patients with any of the following criteria were excluded: transient bacteremia, polymicrobial bacteremia, non-hospitalized patients, patients with do-not-resuscitate orders, and neutropenic patients (absolute neutrophil count < 500/mm^3^). Written informed consent was obtained from all participants. The study was approved by the Ethics Committee of the Hospital Universitario Virgen Macarena (reference number 1578).

Demographic data (including the age, sex, height, and weight of the patient), mode of acquisition of the infection (nosocomial or community-acquired), source of BSI according to clinical and microbiological criteria, serum creatinine concentrations, adverse events potentially related to therapy and 30-day mortality were recorded. Collection of serum creatinine concentrations was standard-of-care, and creatinine clearance was calculated daily using the Cockcroft–Gault equation [28]. Glomerular filtration rate (GFR) was estimated using the Chronic Kidney Disease Epidemiology Collaboration (CKD-EPI) equation [29], the patients were classified according to the different GFR categories used to classify chronic kidney disease (KDIGO) [30]. Bacterial blood isolates were identified by MALDI-TOF, and piperacillin–tazobactam susceptibility testing was performed by the broth microdilution method, following the European Committee on Antimicrobial Susceptibility Testing (EUCAST) methodology. EUCAST breakpoints for piperacillin–tazobactam were used to interpret susceptibility [31].

### 4.2. Antibiotic Therapy

As local dosing recommendations changed in the course of the study period, patients received two different piperacillin–tazobactam regimens: (a) 4/0.5 g (4 h infusion) q8 h, and (b) first dose 4/0.5 g (30 min infusion) followed by 4/0.5 g (4 h infusion) starting immediately after the first dose and then 4/0.5 g (4 h infusion) q8 h, following Roberts et al. with modifications [12]. Patients with creatinine clearance < 20 mL/min/1.73 m^2^ (classified as severely decreased renal clearance or kidney failure) received 4/0.5 g every 12 h, according to the summary of product characteristics (SPC) recommendations [32].

### 4.3. Piperacillin Serum Concentration Assay

Blood samples were taken at steady state, and sampling was performed 1, 4, 6, and 8 h after the commencement of piperacillin infusion, collected in EDTA tubes, centrifuged and stored at −80 °C until analysis. A high-performance liquid chromatography (HPLC) method described by McWhinney et al. was used to analyze piperacillin serum concentrations [33]. FDA guidance on bioanalytical method validation was used to validate the method [34]. The linear range of quantification was 1–500 mg/L. Relative standard deviations and relative errors of the inter- and intra-assay were less than 8.2%, and the lower limit of quantification was 1 mg/L.

### 4.4. Pharmacokinetic Analyses

The nonparametric adaptive grid (NPAG) algorithm embedded in the Pmetrics software package was used to fit a population pharmacokinetic model to the data [35]. For the population pharmacokinetic analysis, one- and two-compartment linear models were fitted to the piperacillin plasma concentration–time data. Covariate model building was performed using sequential assessment of biologically plausible clinical parameters. Forward inclusion was based on the model selection criteria and significant correlation with one of the pharmacokinetic parameters. Creatinine clearance, weight, age, sex, and body mass index (BMI) were explored as covariates for each structural model. The data were weighted by the inverse of the estimated assay variance, which was determined from quality control samples used to estimate inter-day assay variance and given by SD (mg/L) = gamma × (0.4388 + 0.027 × C), where C is the piperacillin concentration, and gamma is an estimate of process noise expressed as multiples of assay variance [35]. The goodness-of-fit of each model to the data was assessed using a combination of the following: (i) log-likelihood values; (ii) coefficients of determination (R2) from linear regression of the observed vs. predicted values before and after the Bayesian step; (iii) minimization of bias and imprecision of the observed vs. predicted plots, (iv) normalized prediction distribution errors (NPDE); (v) the distribution of weighted residual errors; and (vi) a visual predictive check (VPC) plot.

### 4.5. Simulations and Probability of Target Attainment

A 2000-patient Monte Carlo simulation was performed using a semi-parametric sampling method available in Pmetrics [35,36]. The final model consisted of 13 support points. Each point was a set of values for each parameter in the model and the probabilities of those values to predict piperacillin concentrations observed in the population. Each support point then served as the mean for a multivariate normal distribution, weighted by the probability of the point, with covariance equal to the covariance matrix of the full model divided by the number of points (i.e., 13). Semi-parametric sampling from this weighted, multivariate, multimodal normal distribution was used to generate a simulated population of 2000 patients each with their own set of PK parameters. Piperacillin dosages, including short and extended infusions and a loading dose, were simulated as follows: Dosage 1, 4/0.5 g (0.5 h infusion) q8 h; Dosage 2, 4/0.5 g (4 h infusion) q8 h; Dosage 3, first dose 4/0.5 g (0.5 h infusion) followed by 4/0.5 g (4 h infusion) starting immediately after the first dose and then 4/0.5 g (4 h infusion) q8 h. Dose adjustments of piperacillin in patients with severely decreased renal clearance (15–29 mL/min/1.73 m^2^) and kidney failure (<15 mL/min/1.73 m^2^) were simulated with the aim of optimizing piperacillin pharmacodynamics in these patient subgroups. The following piperacillin dosages, which included standard short (0.5 h) and extended (4 h) infusion, were simulated: Dosage 1, 4/0.5 g (0.5 h infusion) q12 h; Dosage 2, 4/0.5 g (4 h infusion) q12 h; Dosage 3, first dose [4/0.5 g (0.5 h infusion) + 4/0.5 g (4 h infusion)] followed by 4 g (4 h infusion) q12 h. For the probability of pharmacodynamic target attainment (PTA) analysis, MICs ranging between 0.0625 and 256 mg/L were assessed in doubling dilutions, using EUCAST susceptibility breakpoints (susceptible ≤ 8 mg/L and resistant > 16 mg/L), and a pharmacodynamic index to predict efficacy, *f*T_MIC_ ≥ 50% of the dosing interval, was used [37]. The unbound fraction of piperacillin was fixed at 0.7 [38]. A PTA of ≥ 90% was considered optimal.

### 4.6. Toxicodynamic Analysis

The probability of reaching neurotoxicity- (C_min_ piperacillin steady-state concentrations above 361.4 mg/L or 157.2 mg/L) and nephrotoxicity- (C_min_ piperacillin steady-state concentrations above 452.65 mg/L) related thresholds, respectively, was analyzed over a 24 h interval (day 2) [15,16]. For these analyses, the unbound piperacillin concentration was used.

## 5. Conclusions

In conclusion, given the association between early and appropriate antibiotic therapy and improved clinical outcomes for critically ill patients [1,4,39,40], our data support the use of extended piperacillin infusions early in patients with invasive infections caused by Enterobacteriaceae, even if the patients are not critically ill and have a low risk of neurotoxicity or nephrotoxicity events. The benefits of a loading dose are not clear and should be explored in other populations such as patients with augmented renal clearance. These findings should be confirmed in other studies with similar populations and by using clinical endpoints.

## Figures and Tables

**Table 1:** Baseline demographic and clinical characteristics of 27 patients with bloodstream infections due to Enterobacteriaceae, treated with piperacillin–tazobactam (TZP). Data are number of patients (percentage), unless otherwise stated. CrCl, creatinine clearance, Sequential Organ Failure Assessment (SOFA), ESBL, extended-spectrum beta-lactamase, MIC,

Variable	No. of Cases (%) Unless Otherwise Stated
Male gender	17 (62.96)
Age in years, median (range)	76.5 (48–86)
Body mass index ≥ 25	19 (79.1)
CrCl in mL/min, median (range)	50.7 (45.3–255.3)
Charlson score, median (range)	2.5 (0–8)
*Comorbidities:*	
Diabetes mellitus	13 (48.1)
Chronic pulmonary disease	4 (14.8)
Cancer	11 (40.7)
Liver cirrhosis	1 (3.7)
Immunosuppressive therapy	1 (3.7)
Source of bacteremia	
Urinary tract	18 (66.67)
Biliary tract	7 (25.9)
Other intraabdominal infection	2 (7.4)
Community-acquired bacteremia	11 (40.7)
Pitt score, median (range)	2 (0–5)
SOFA score at diagnosis, median (range)	3 (0–8)
*Microorganism:*	
*Escherichia coli*	15 (55.5)
*Klebsiella oxytoca*	6 (22.2)
*Klebsiella pneumoniae*	3 (11.1)
*Enterobacter aerogenes*	2 (7.4)
*Enterobacter cloacae*	1 (3.7)
ESBL producer	3 (11.1)
Hours from blood culture until first TZP dose, median (range)	1.6 (0–11)
MIC	25 (92.5)
1 mg/L	3 (12)
2 mg/L	13 (52)
4 mg/L	5 (20) ^1^
8 mg/L	3 (12) ^2^
16 mg/L	0
>16 mg/L	1 (3.7)
*Outcome:*	
Lack of improvement, day 2	5 (18.5)
Clinical failure, day 14	4 (14.8)
Mortality, day 30	1 (3.7)

^1^ 2 were ESBL producers. ^2^ 1 was an ESBL producer.

**Table 2:** Final population pharmacokinetic parameter estimates for 27 patients with bloodstream infections due to Enterobacteriaceae treated with piperacillin–tazobactam.

Parameter	Mean	SD	Median
Drug Clearance, CL (L/h) CL = Intercept + slope × creatinine clearance (L/h)			
Intercept (L/h) ^1^	4.556	5.035	3.503
Slope	1.353	1.032	1.39
Volume of distribution, V_c_ (L)	30.68	23.349	20.039

^1^ Intercept and slope are the coefficients of a linear relationship of piperacillin clearance versus ClCr.

**Table 3:** Probability of target attainment (PTA) using an *f*TMIC_0–24h_ = 50% as pharmacodynamic target after simulation in patients with normal or severe renal impairment according to KDIGO clinical practice guideline for acute kidney injury: Dosage 1, 4 g (0.5 h infusion) q8 h; Dosage 2, 4 g (4 h infusion) q8 h; Dosage 3, first dose [4 g (0.5 h infusion) + 4 g (4 h infusion)] followed by 4 g (4 h infusion) q8 h. Dosage 4, 4 g (0.5 h infusion) q12 h; Dosage 5, 4 g (4 h infusion) q12 h; Dosage 6, first dose [4 g (0.5 h infusion) + 4 g (4 h infusion)] followed by 4 g (4 h infusion) q12 h. Green, yellow, and red boxes indicate piperacillin PTA ≥ 90%, <90- ≥50%, and <50%, respectively.

Target *f*T > MIC = 50%	Normal (90–129 mL/min/1.73 m^2^)			Severely Decreased (15–29 mL/min/1.73 m^2^) Kidney Failure (<15 mL/min/1.73 m^2^)		
	Dosage			Dosage		
MIC (mg/L)	1	2	3	4	5	6
0.06	99.8	100	100	99.4	99.9	100
0.125	99.2	100	100	99.2	99.9	100
0.25	99	100	100	99.2	99.9	100
0.5	98.8	100	100	99.2	99.8	100
1	98.4	100	100	98.9	99.7	100
2	97.4	100	100	98.8	99.6	100
4	93.4	100	100	98.5	99.5	99.9
8 EUCAST (S)	76.4	100	100	92.9	96.1	99.7
16 EUCAST (I)	56.8	95.8	94.6	77.3	90.7	94.3
32 (EUCAST R)	14.2	28	45.8	38.7	61.1	81.3
64	1.2	2	8.4	6.9	11.7	31.8
128	0	0	0.2	1.2	1.2	4.6
256	0	0	0	0.2	0.2	0.7

## Data Availability

The data presented in this study are available on request from the corresponding author.
